# Effect of MACC1 Genetic Polymorphisms and Environmental Risk Factors in the Occurrence of Oral Squamous Cell Carcinoma

**DOI:** 10.3390/jpm11060490

**Published:** 2021-05-31

**Authors:** Rei-Hsing Hu, Chun-Yi Chuang, Chiao-Wen Lin, Shih-Chi Su, Lun-Ching Chang, Ssu-Wei Wu, Yu-Fan Liu, Shun-Fa Yang

**Affiliations:** 1 Department of Biomedical Sciences, Chung Shan Medical University, Taichung 402, Taiwan; hrexing@gmail.com; 2 School of Medicine, Chung Shan Medical University, Taichung 402, Taiwan; cyi4602@gmail.com; 3 Department of Otolaryngology, Chung Shan Medical University Hospital, Taichung 402, Taiwan; 4 Institute of Oral Sciences, Chung Shan Medical University, Taichung 402, Taiwan; cwlin@csmu.edu.tw; 5 Department of Dentistry, Chung Shan Medical University Hospital, Taichung 402, Taiwan; 6 Whole-Genome Research Core Laboratory of Human Diseases, Chang Gung Memorial Hospital, Keelung 204, Taiwan; ssu1@cgmh.org.tw; 7 Department of Dermatology, Drug Hypersensitivity Clinical and Research Center, Chang Gung Memorial Hospital, Linkou 333, Taiwan; 8 Department of Mathematical Sciences, Florida Atlantic University, Boca Raton, FL 33431, USA; changl@fau.edu; 9 Institute of Medicine, Chung Shan Medical University, Taichung 402, Taiwan; richman929@hotmail.com; 10 Department of Pediatrics, Chung Shan Medical University Hospital, Taichung 402, Taiwan; 11 Department of Medical Research, Chung Shan Medical University Hospital, Taichung 402, Taiwan

**Keywords:** SNPs, MACC1, OSCC, genotyping, betel qui d chewing

## Abstract

MACC1 (Metastasis Associated in Colon Cancer 1) is found to regulate the hepatocyte growth factor (HGF)/Met signal pathway, and plays an important role in tumor proliferation, angiogenesis, and metastasis. However, the relationships between *MACC1* SNPs (single nucleotide polymorphisms) and oral cancer are still blurred. In this study, five SNPs (rs3095007, rs1990172, rs4721888, rs975263, and rs3735615) were genotyped in 911 oral cancer patients and 1200 healthy individuals by real-time polymerase chain reaction (PCR), and the associations of oral cancer with the SNP genotypes, environmental risk factors, and clinicopathological characteristics were further analyzed. Our results showed that individuals who had GC genotype or C-allele (GC + CC) in rs4721888 would have a higher risk for oral cancer incidence than GG genotype after adjustment for betel quid chewing, cigarette smoking, and alcohol drinking. Moreover, the 715 oral cancer patients with a betel quid chewing habit, who had C-allele (TC + CC) in rs975263, would have a higher risk for lymph node metastasis. Further analyses of the sequences of rs4721888 revealed that the C-allele of rs4721888 would be a putative exonic splicing enhancer. In conclusion, *MACC1* SNP rs4721888 would elevate the susceptibility for oral cancer, and SNP rs975263 would increase the metastasis risk for oral cancer patients with a betel quid chewing habit. Our data suggest that SNP rs4721888 could be a putative genetic marker for oral cancer, and SNP rs975362 may have the potential to be a prognostic marker of metastasis in an oral cancer patient.

## 1. Introduction

According to the global cancer statistics 2018, of the 18.1 million global new cases of all cancers, oral cancer is around 2.0% and its death is around 1.9% of the 9.6 million cancer deaths [1]. In Taiwan, the incidence of oral cancer was 21.98 per 100,000 persons in 2017. Oral cancers, one of the head and neck cancers (HNCs), are highly aggressive malignancies with a high mortality rate, and the five-year survival rate of the distant stage is lower than 50%. Most oral cancers belong to the oral squamous cell carcinoma (OSCC) category, and around 25–40% of OSCCs are commonly tongue squamous cell carcinomas (TSCCs) [2]. OSCC is also one of the most common tumors of the head and neck region, and it is an undifferentiated nonkeratinizing carcinoma in the histological aspect with a high potential for metastasis. The factors associated with OSCC are human papillomavirus infection [3,4,5,6], and ordinary cultural habits such as smoking, drinking alcohol, and betel quid chewing, which is a popular risk for oral tumorigenesis in south-east Asian countries [7].

Metastasis associated with the colon cancer-1 (MACC1) gene, which plays an important role in regulating the hepatocyte growth factor (HGF)/MET signaling pathway, is recognized as a prognostic biomarker for colon cancer [8]. The HGF/MET signaling pathway will activate several signal pathways, such as the extracellular signal-regulated kinases/ mitogen-activated protein kinase (ERK/MAPK) pathway for cell proliferation, the PI3K/Akt pathway for cell survival, and others for epithelial-mesenchymal transition (EMT), cell motility, invasion, and metastasis [9]. In addition, the overexpression of MACC1 is very important for tumor metastases and poor prognosis [10]. Other than colorectal cancer (CRC), MACC1 is also considered a biomarker for several other cancers [11,12,13,14,15]. In HNC studies, Li et al. found that MACC1 overexpression was highly correlated with poor overall patient survival of TSCC, and the knockdown of MACC1 expression in TSCCA cells obviously downregulated the migration, invasion, and resistance to cisplatin [16]. Furthermore, Meng et al. reported that MACC1 was also significantly correlated with the clinical stage in patients with nasopharyngeal carcinoma (NPC) and the knockdown of MACC1 expression in NPC cells also inhibited the cell proliferation, migration, and invasion [17]. Nonetheless, it still needs more studies on the relationship between oral cancer and MACC1.

In the previous study, Schmid et al. found that the single nucleotide polymorphism (SNP) in the *MACC1* gene, rs975263, might be related to decreased metastasis-free survival for younger colon cancer patients with the TC genotype in early stages [18]. Besides this, CRC patients with a G-allele of SNP rs1990172 showed an obviously reduced overall survival [19]. In the research of hepatocellular carcinoma (HCC), SNPs rs1990172 and rs975263 could be potential biomarkers for recurrence in HCC patients after liver transplantation [20]. Moreover, the HCC patients among smokers with rs1990172 “CA + AA” variants had a lower risk of forming large tumors, and patients among drinkers with rs4721888 “GC + CC” variants had a higher risk of vascular invasion, and patients with rs975263 “TC + CC” variants had a significantly abnormal AST/ALT ratio before adjustment for age and drinking [21]. These results demonstrated that the SNP of *MACC1* may be a biomarker for evaluating the progression and recurrence in HCC patients. In breast cancer, the human epidermal growth factor 2 (HER2)-positive patients with a G-allele of rs1990172 or T-allele of rs975263 had a lower survival rate, but the patients with a C-allele of rs3735615 had a better survival rate [22]. Furthermore, in uterine cervical cancers of Taiwanese female patients with a CC genotype of rs975263 had a higher risk for vaginal invasion [23].

Recently, more evidence has been disclosed on the relationships between *MACC1* SNPs and carcinoma as in the aforementioned results. Nonetheless, the relationships of *MACC1* SNPs with clinical variables, clinical outcomes, and the development of oral cancer have not been studied. Therefore, we conducted studies on five *MACC1* SNPs, rs3095007, rs1990172, rs4721888, rs975263, and rs3735615 to investigate their diagnosable benefits of oral cancer patients in aspects of the disease process, the prognosis, and the clinicopathological characteristics in central Taiwan.

## 2. Materials and Methods

### 2.1. Study Population

The 911 male patients with OSCC, who were undergoing tumor resection, were recruited from 2007 to 2016 from Changhua Christian Hospital and Chung Shan Medical University Hospital as a study group. Due to the unique epidemiology of oral cancer in Taiwan (less than 5% cases are female), the inclusion criteria were (1) diagnosed with oral cancers, (2) age ≥ 20 years, (3) of male gender, and (4) were followed up with for at least six months. We selected 1200 healthy male individuals that had no self-reported history of cancer of any sites and were without oral precancerous lesions from the Taiwan Biobank as the control group. The information of the exposure to environmental risk factors, such as cigarette smoking, alcohol drinking, and betel quid chewing, was harvested with a questionnaire for the patients, and the aforementioned information of the control group was obtained from the record of Taiwan Biobank. The information on the oral cancer, such as tumor size, neck lymph node metastasis, distant metastasis, and cell differentiation grade, was obtained from patients’ medical records. Each enrolled participant provided the written informed consent before their specimens and medical information were collected for this study. The research design was approved by the Institutional Review Board of Chung Shan Medical University Hospital (CSMUH No: CS15125).

### 2.2. MACC1 SNP Selection and DNA Extraction and Genotyping

To obtain adequate power for evaluating the potential association, we investigated the SNPs rs3095007, rs1990172, rs4721888, rs975263, and rs3735615 in *MACC1*, those with minor allele frequencies ≥ 5% and also reported as involved in the susceptibility to the development of various cancer types [21,23]. The specimens from oral cancer patients and normal controls were further isolated from the genomic DNA with QIAamp DNA blood mini kits. From the whole blood specimens, the buffy coats were isolated and then the genomic DNA was extracted according to the protocol in previous studies [24,25,26]. The *MACC1* rs3095007 (assay IDs: C__27452671_10), rs1990172 (assay IDs: C___2632417_10), rs4721888 (assay IDs: C__27869756_10), rs975263 (assay IDs: C___2632422_20), and rs3735615 (assay IDs: C__27489506_10) SNP was carried out by using the TaqMan™ Genotyping Master Mix on the ABI StepOne™ Real-Time PCR System (Applied Biosystems, Foster City, CA, USA), and the genotypes were determined using SDS software (version 3.0) (Applied Biosystems, Foster City, CA, USA) as in the previous studies [21,27]. The context sequence and representative figure of the Taqman assay are presented in Table S1 and Figure S1.

### 2.3. Statistical Analysis

In the two groups of Patients and Controls, Chi-Square or Fisher tests were applied for the categorical variables, and the Mann–Whitney U test was applied for the continuous variables. The numbers of each genotype of *MACC1* in both groups were also tested using the Chi-Square test. Hardy–Weinberg equilibrium with biallelic marker was tested using the Chi-Square goodness-of-fit test, and the pairwise linkage disequilibrium of the SNPs genotype was analyzed with Haptoview software. The odds ratio (OR), adjusted odds ratio (AOR), and their 95% confidence intervals (95% CIs) were calculated by using the multiple logistic regression model with adjustments for the risk factors, which are age, betel quid chewing, alcohol drinking, and cigarette smoking, and the values were further analyzed using SPSS statistical software (SPSS, Chicago, IL, USA) to evaluate the significant difference with *p* < 0.05.

## 3. Results

### 3.1. Clinicopathological Characteristics of OSCC Patients

The clinical parameters of hospital-based case-control study were conducted to explore (Table 1). There was no statistical difference of age distribution between normal control men and patients with oral cancer (*p* = 0.538). In the aspect of carcinogenic substances and living habits, 78.5% of people were used to betel quid chewing in the patient group, but only 16.6% in the control group (*p* < 0.001); 88.8% of people were used to cigarette smoking in the patient group, and 53% in the control group (*p* < 0.001); 53.8% of people were used to alcohol drinking in the patient group, and 19.7% in the control group (*p* < 0.001). According to this data, betel quid chewing, alcohol drinking, and cigarette smoking habits were significantly associated with OSCC (Table 1). In the patient group, from the clinicopathological distributions, the proportions of early stage (stage I and II) and late stage (stage III and IV) were similar, 49.5% vs. 50.5%. For the tumor size (tumor T status), patients with less than 4-cm sized tumors (T1 + T2) were 57.7% of the patient group, and the amount of T3 + T4 was 42.3%. In the aspect of regional lymph node metastasis status, around two-thirds of the patients belonged to N0 (67.7%), the remaining 32.3% patients had cervical lymphatic metastasis (N1 + N2 + N3). In addition, for the metastasis status, there were nine patients with distant metastasis (M1), the proportion was less than 1% in the patient group. From the histopathological reports of cell differentiation, 85.9% of the patients had moderate or poor cell differentiation (Table 1).

### 3.2. Association of MACC1 Genetic Variants with the Incidence of OSCC

We validated the allele frequency of rs3095007, rs1990172, rs4721888, rs975263, and rs3735615 with high-quality catalogs of variation such as NCBI dbGaP [28], 1000 Genomes Project [29], and gnomAD-Genome [30], the distribution and effect of human genetic variation were similar. We estimated the odds ratios (ORs) to study the association of the five *MACC1* independent SNPs with oral cancer incidence by logistic regression models. There were no obvious correlations between the incidences of oral cancer and *MACC1* SNPs when comparing the genotype frequencies of the 5 *MACC1* SNPs in the case-control study. On adjusting the ORs (AORs) with a 95% confidence interval (CI) for risk factors including betel nut chewing, alcohol, and tobacco consumption, compared to the wild-type *MACC1* SNP rs4721888 GG genotype, the GC genotype had a 1.272-fold (*p* = 0.044, 95% CI = 1.007–1.607) higher risk and was significantly associated with oral cancer as well as C-allele (GC + CC) 1.292-fold (*p* = 0.025, 95% CI = 1.033–1.617) by multiple logistic regression models. Except for *MACC1* SNP rs4721888, there was no statistical difference observed after controlling for the risk factors in other SNPs (Table 2).

### 3.3. MACC1 Genetic Polymorphism and Environmental Risk Factor Betel Quid Chewing

Since we know that betel quid ingredients are an important environmental risk factor for carcinogenicity, we further analyzed them in combination with a genetic variant in oral cancer incidence for the population that had the betel quid chewing habit. There were 199 and 715 individuals in the control and patient groups, respectively. Only *MACC1* SNP rs4721888 was statistically significant among the five *MACC1* SNPs. The estimated ORs were found be higher for the patients with the GC genotype (*p* = 0.027, OR = 1.462, 95% CI = 1.045–2.046) and the C-allele (GC + CC) (*p* = 0.021, OR = 1.457, 95% CI = 1.058–2.005) of *MACC1* non-synonymous SNP rs4721888 was higher than those people with the wild-type GG genotype (Table 3). Even after the adjustment for risk factors, the *MACC1* SNP rs4721888 disclosed a significantly statistical result, the GC genotype (*p* = 0.044, AOR = 1.466, 95% CI = 1.045–2.057) and C-allele (GC + CC) (*p* = 0.020, AOR = 1.466, 95% CI = 1.062–2.024) showed a significant association with the incidence of oral cancers in people who were used to chewing betel quid. Patients with a polymorphic C allele of *MACC1* rs4721888 tended to have an increased risk of oral cancer among those who had a betel quid chewing habit (Table 3). However, no statistical difference was observed in the people who were not used to chewing betel quid (Table 4). There were no statistical differences between the cigarette smoking patients group (Table S2) and the alcohol drinking patients group (Table S3).

**Figure 1:**
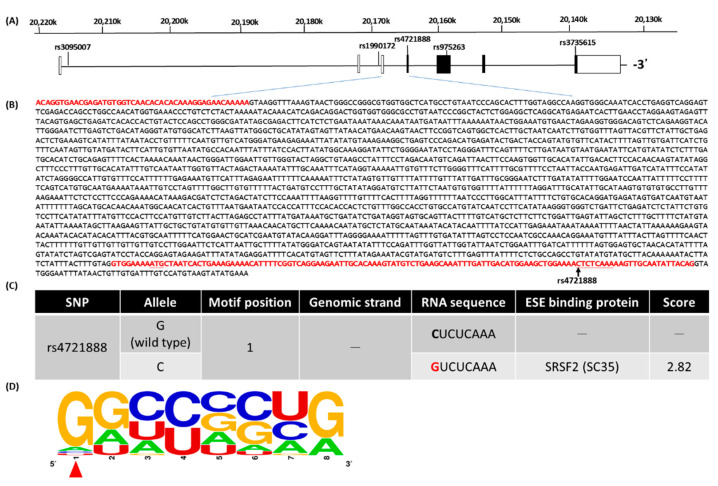
Exonic splicing enhancer binding site polymorphism from SNP rs4721888 [G/C] in the fourth exon of human MACC1 mRNA to increase oral cancer susceptibility among the Taiwan OSCC population. (**A**) The seven exons and six introns in the structure of the MACC1 gene, in addition to the longest open reading frame (ORF), are shown by the filled boxes for the chromosome positions (Chr.7, reference genome GRCh38.p7). There are five selected SNPs including rs3095007, rs1990172, rs4721888, rs975263, and rs3735615 in this study. (**B**) The detailed third and fourth exon-intron sequence of the human MACC1 (NM_182762.3) gene, highlighting the exon sequences with a red font. The underline and arrow indicate the translational start site and SNP rs4721888, respectively. (**C**) Prediction of the putative exonic splicing enhancer (ESE) by using the ESEfinder 3.0. The matrices are based on frequency values by functional systematic evolution of human SR protein motifs of SRSF2 (SC35). The red character indicates the position of the SNP rs4721888 C allele. (**D**) The binding motif of SC35 was created by WEBLOGO. The red arrow indicates the SNP rs4721888 [G/C] alleles that may cause the coding strand sequence to become a putative SC35 ESE binding motif.

We further analyzed the sequences of the G- or C-allele of rs4721888 using the ESEfinder 3.0 (http://krainer01.cshl.edu/cgi-bin/tools/ESE3) to find the putative exonic splicing enhancer (ESE). Interestingly, the C-allele of rs4721888 would be a putative ESE (Figure 1C), which might be recognized by the human-specific serine/arginine-rich (SR) proteins, SC35 protein (Figure 1D).

### 3.4. The Effect of SNP rs975263 on Oral Cancer Patients with Betel Quid Chewing

In order to realize the effects of the five genetic variants in *MACC1* SNPs on oral cancer patients with betel quid chewing, we analyzed if the *MACC1* SNPs had relevance to the patients’ clinicopathological characteristics. For a total of 715 patients with oral cancer, most of the *MACC1* SNPs did not show an association with the characteristics, but the patients with non-synonymous SNP rs975263 showed a significant association with the clinicopathological characteristics. There were 488 patients with wild-type TT genotype of rs975263, and 227 patients with C allele (TC and CC genotypes). From the statistical results, patients with C allele of rs975263 had higher risks for lymph node metastasis (*p* = 0.034, OR = 1.431, 95% CI = 1.026–1.996) than patients with the TT genotype (Table 5). Nevertheless, other characteristics, such as clinical stage, tumor size, and cell differentiation, were not related to the genotypes of rs975263.

**Figure 2:**
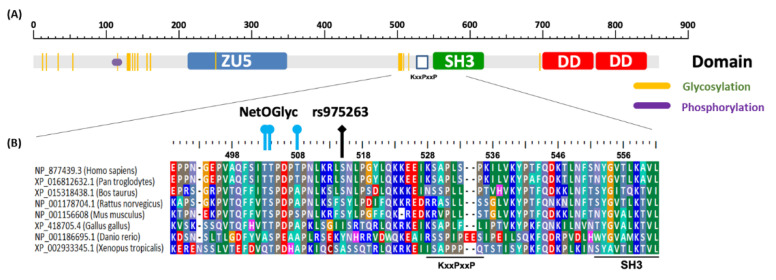
The diagram of putative MACC1 protein domains and predicted post-translational modification sites. (**A**) The human MACC1 protein was analyzed by using the GlycoDomainViewer. The grey bar indicates the MACC1 853 amino acids and different domains, the blue rectangle indicates the ZO-1 and Unc5-like netrin receptors domain (ZU5), the green rectangle indicates Src Homology 3 (SH3), and the red rectangle indicates the death domain (DD). The orange lines show the putative glycosylation sites and purple dots show the predicted phosphorylation residues (S112, S115, S116). (**B**) The rs975263 regions of the selected organisms were aligned, the glycosylation sites were predicted by NetOGlyc 3.0 Server and shown as lines above the sequences.

We did not find the effect of different genotypes on splicing, but we found that the serine 515 (wild-type rs975263, T allele) would be a putative glycosylation site by using NetOGlyc 3.0 (http://www.cbs.dtu.dk/services/NetOGlyc-3.0/). Furthermore, from the phosphorylation prediction website PhosphoSitePlus, we also found that S515 could be a putative phosphorylation site. The C allele of rs975263 would change serine to leucine, the glycosylation site would be eliminated by NetOGly 3.0, and the phosphorylation might disappear since leucine is rarely reported as a phosphorylation residue (Figure 2A).

## 4. Discussion

The risk factors, cigarette smoking, alcohol smoking, and betel quid chewing, are generally acknowledged as environmental carcinogens [31]. Similar results were also observed in this study, the ratio of cigarette smoking in the control group was less than 20%, but in the patient group, it was close to 90%. The ratio of alcohol drinking in the controls was less than 20%, but in the patient group, it was more than half. The ratio of betel chewing in the control group was around 20%, but in the patient group, it was close to 80%. Therefore, the distributions of the individuals in the study corresponded with previous studies. In Taiwan, the culture for chewing betel quid is popular, and also in India and Southeast Asian countries, and there are many reports on the carcinogenesis mechanisms of the ingredients of betel nuts, and the involved cellular signal pathways are Pi3k/Akt, NF-κB, TNF-β/Smad, GSK-3β, and so on [32]. Besides this, the components of betel nut may induce multiple pathways, such as reactive oxygen species (ROS), epidermal growth factor (EGF)/epidermal growth factor receptor (EGFR), a disintegrin and metalloproteinases (ADAMs), Janus kinase (JAK), Src, mitogen-activated protein kinase (MEK)/extracellular signal-regulated kinase (ERK), cytochrome p450 A1 (CYPA1), and cyclooxygenase (COX), and could cause the abnormality of cell cycles and differentiation [33].

Recently, there are several reports on the relationship between *MACC1* SNPs and cancers, such as colorectal cancer, hepatocellular carcinoma, and breast cancer. Here, we disclosed the relationship of oral cancer and *MACC1* SNPs and found that people with GC genotype or C-allele genotype in rs4721888 would have a higher risk of oral cancer incidence whether the risk factors were adjusted or not. Dai et al. also found that Chinese Han women with the GC or CC genotype in rs4721888 had higher susceptibility for breast cancer [34]. Compared to the aforementioned report, the HCC patients with alcohol drinking, who had the GC or CC variants in rs4721888, had a higher risk of vascular invasion [21]. Therefore, *MACC1* SNP rs4721888 might have some effect on cancer incidence or susceptibility. It also influences the splicing of MACC1 mRNA (Figure 1B) and might cause different accumulation quantities of MACC1 mRNA and/or protein between the two genotypes of G- or C-allele of rs4721888. According to the AceView database of NCBI [35], which provides collected experimental cDNA sequences, there are 16 types of MACC1 mRNAs, and only three types contain the exon 4, which is rs4721888 [35]. This might imply that the G-allele of rs4721888 would cause lesser functionality in MACC1 mRNA and/or protein than the C-allele of rs4721888 does (Figure 1C). Considering the previous studies, a higher expression or accumulation of MACC1 protein would induce tumor progression and tumorigenicity [17,36], thus, this may be a practicable direction to confirm the expression or accumulation quantities of MACC1 of different rs4721888 genotypes for the tumorigenesis of oral cancer.

Because MACC1 is correlated with cancer metastasis, we also analyzed the relationships between the genotypes in the 5 *MACC1* SNPs of oral cancer patients with betel quid chewing habits and their clinicopathological reports. Only TC + CC genotype of SNP rs975263 showed significance for the lymph node metastasis in oral cancer patients with betel quid chewing habits. This result is similar to the aforementioned studies of rs975263 in younger colon cancer patients with TC genotype decreasing metastasis-free survival [18], and uterine cervical cancers of Taiwanese female patients with CC genotype suffering a higher risk of vaginal invasion [23]. However, in the HER2-positive breast cancer patients, the TT genotype of rs975263 is related to a lower survival rate [22]. We supposed that the T allele of *MACC1* SNP rs975263 might cause the MACC1 proteins with different post-translational modifications (PTMs), glycosylation or phosphorylation, to play a reciprocal switch role in regulating the cellular location or biofunction of MACC1, that is, c-myc, RNA polymerase II, estrogen receptor β, etc. [37,38,39]. Since the *O*-linked beta-*N*-acetylglucosamine (*O*-GlcNAc) glycosylation and phosphorylation may be a switch to regulate the transcription factors, and S515 is not in the conserved functional domain, ZU5 and SH3 domain, and is conservative in mammalian species (Figure 2B), it could be a potential regulatory site [40]. Although many reports clarified the importance of PTMs, such as glycosylation, phosphorylation, ubiquitination, methylation, and so on, for inflammation or cancer [41,42], it still needs more studies on the PTMs of MACC1.

Here, we reported the relationships between oral cancer and *MACC1* SNPs, people who had rs4721888 variants may have more susceptibility to oral cancer whether they were used to betel quid chewing or not, and the oral cancer patients who had the habit of betel quid chewing and rs975263 variants would have more potential risk of lymph node metastasis. This study also revealed the relationships between environmental risk factors and oral cancer, moreover, the C-allele of rs4721888 might be a diagnosis and prevention genetic marker for early-stage oral cancer, and the C-allele of rs975263 might be further studied to be a prognostic biomarker for oral cancer progression, therapy, and prognosis.

## 5. Conclusions

This study is the first research on the effect of *MACC1* SNPs with oral cancer, and we also found that people with GC genotype or C-allele in rs4721888 would have a higher risk of oral cancer susceptibility than GG genotype. Besides this, people who had the habit of betel quid chewing and GC genotype or C-allele genotype of rs4721888 would also have a higher susceptibility to oral cancer incidence. Moreover, oral cancer patients with a betel quid chewing habit, who also had the TC genotype or C-allele in rs975263, would have a higher risk of lymph node metastasis. The result proved the synergistic effect of the *MACC1* SNPs, and environmental risk factors for oral cancer, and could be further studied to apply the rs4721888 genetic marker for diagnosis and prevention and to develop the rs975263 genetic marker for prognosis.

## Figures and Tables

**Table 1:** The distributions of demographical characteristics and clinical parameters in 1200 controls and 911 patients with OSCC.

Variable	Controls (*n* = 1200)	Patients (*n* = 911)	*p* Value ^♀^
Age (yrs)	53.9 ± 10.0	55.1 ± 11.0	
<55	566 (47.2%)	442 (48.5%)	*p* = 0.538
≥55	634 (52.8%)	469 (51.5%)	
Betel quid chewing			
No	1001 (83.4%)	196 (21.4%)	*p* < 0.001 *
Yes	199 (16.6%)	715 (78.5%)	
Cigarette smoking			
No	564 (47.0%)	102 (11.2%)	*p* < 0.001 *
Yes	636 (53.0%)	809 (88.8%)	
Alcohol drinking			
No	963 (80.3%)	421 (46.2%)	*p* < 0.001 *
Yes	237 (19.7%)	490 (53.8%)	
Stage			
I		245 (26.9%)	
II		206 (22.6%)	
III		78 (8.6%)	
IV		382 (41.9%)	
Tumor T status			
T1		273 (30.0%)	
T2		253 (27.7%)	
T3		72 (7.9%)	
T4		313 (34.4%)	
Lymph node status			
N0		617 (67.7%)	
N1		90 (9.9%)	
N2		168 (18.4%)	
N3		36 (4.0%)	
Distant metastasis			
M0		902 (99.0%)	
M1		9 (1.0%)	
Cell differentiation			
Well		128 (14.1%)	
Moderate		738 (81.0%)	
Poor		45 (4.9%)	

♀ Mann–Whitney U test was used between healthy controls and patients with OSCC. * *p*-value < 0.05 as statistically significant. Betel quid chewing and alcohol drinking are defined as the behavioral use of betel quid and alcoholic drinking, respectively. Cigarette smoking is defined as the current smoking of at least one cigarette per day during the last three months.

**Table 2:** The distribution of genotypic frequencies in *MACC1* SNPs in case-control groups.

Variable	Controls (%)	Patients (%)	OR ^a^ (95% CI)	AOR ^b^ (95% CI)
rs3095007				
GG	1003 (83.6%)	764 (83.9%)	1.00	1.00
GT	188 (15.7%)	142 (15.6%)	0.992 (0.782–1.257)	0.908 (0.668–1.233)
TT	9 (0.7%)	5 (0.5%)	0.729 (0.243–2.185)	0.470 (0.121–1.820)
GT + TT	197 (16.4%)	147 (16.1%)	0.980 (0.776–1.237)	0.883 (0.654–1.193)
rs1990172				
GG	892 (74.3%)	683 (75.0%)	1.00	1.00
GT	294 (24.5%)	208 (22.8%)	0.924 (0.754–1.133)	0.853 (0.657–1.109)
TT	14 (1.2%)	20 (2.2%)	1.866 (0.936–3.721)	2.021 (0.823–4.961)
GT + TT	308 (25.7%)	228 (25.0%)	0.967 (0.793–1.179)	0.900 (0.697–1.163)
rs4721888				
GG	634 (52.8%)	460 (50.5%)	1.00	1.00
GC	484 (40.3%)	382 (41.9%)	1.088 (0.909–1.302)	1.272 (1.007–1.607) *p* = 0.044
CC	82 (6.9%)	69 (7.6%)	1.160 (0.824–1.633)	1.414 (0.911–2.196)
GC + CC	566 (47.2%)	451 (49.5%)	1.098 (0.924–1.305)	1.292 (1.033–1.617) *p* = 0.025
rs975263				
TT	820 (68.3%)	623 (68.4%)	1.00	1.00
TC	350 (29.2%)	260 (28.5%)	0.978 (0.808–1.184)	1.008 (0.787–1.290)
CC	30 (2.5%)	28 (3.1%)	1.228 (0.726–2.078)	1.079 (0.550–2.116)
TC + CC	380 (31.7%)	288 (31.6%)	0.998 (0.829–1.201)	1.014 (0.798–1.288)
rs3735615				
GG	866 (72.2%)	655 (71.9%)	1.00	1.00
GC	311 (25.9%)	238 (26.1%)	1.012 (0.831–1.232)	0.849 (0.659–1.096)
CC	23 (1.9%)	18 (2.0%)	1.035 (0.554–1.933)	0.797 (0.356–1.786)
GC + CC	334 (27.8%)	256 (28.1%)	1.013 (0.836–1.228)	0.846 (0.660–1.084)

^a^ The odds ratios (ORs) with 95% confidence intervals were estimated by logistic regression models. ^b^ The adjusted odds ratios (AORs) with 95% confidence intervals were estimated by multiple logistic regression models after controlling for betel nut chewing, alcohol, and tobacco consumption.

**Table 3:** The *MACC1* SNPs genotype frequencies of people who were used to chewing betel quid.

Variable	Controls (N = 199) *n* (%)	Patients (N = 715) *n* (%)	OR ^a^ (95% CI)	AOR ^b^ (95% CI)
rs3095007				
GG	163 (81.9%)	595 (83.2%)	1.00	1.00
GT	34 (17.1%)	115 (16.1%)	0.927 (0.609–1.410)	0.930 (0.608–1.421)
TT	2 (1.0%)	5 (0.7%)	0.685 (0.132–3.562)	0.687 (0.129–3.650)
GT + TT	36 (18.1%)	120 (16.8%)	0.913 (0.606–1.377)	0.916 (0.605–1.388)
rs1990172				
GG	137 (68.8%)	539 (75.4%)	1.00	1.00
GT	60 (30.2%)	160 (22.4%)	0.678 (0.447–0.963)	0.686 (0.481–0.978)
TT	2 (1.0%)	16 (2.2%)	2.033 (0.462–8.949)	2.020 (0.455–8.970)
GT + TT	62 (31.2%)	176 (24.6%)	0.722 (0.511–1.019)	0.729 (0.515–1.033)
rs4721888				
GG	120 (60.3%)	365 (51.0%)	1.00	1.00
GC	67 (33.7%)	298 (41.7%)	1.462 (1.045–2.046) *p* = 0.027	1.466 (1.045–2.057) *p* = 0.044
CC	12 (6.0%)	52 (7.3%)	1.425 (0.736–2.758)	1.463 (0.750–2.853)
GC + CC	79 (39.7%)	350 (49.0%)	1.457 (1.058–2.005) *p* = 0.021	1.466 (1.062–2.024) *p* = 0.020
rs975263				
TT	135 (67.8%)	488 (68.3%)	1.00	1.00
TC	58 (29.1%)	203 (28.4%)	0.968 (0.683–1.372)	0.977 (0.687–1.389)
CC	6 (3.0%)	24 (3.4%)	1.107 (0.443–2.762)	1.178 (0.467–2.972)
TC + CC	64 (32.2%)	227 (31.7%)	0.981 (0.701–1.374)	0.995 (0.708–1.399)
rs3735615				
GG	131 (65.8%)	507 (70.9%)	1.00	1.00
GC	63 (31.7%)	192 (26.9%)	0.787 (0.559–1.110)	0.805 (0.569–1.138)
CC	5 (2.5%)	16 (2.2%)	0.827 (0.297–2.298)	0.796 (0.278–2.278)
GC + CC	68 (34.2%)	208 (29.1%)	0.790 (0.566–1.104)	0.804 (0.574–1.128)

^a^ The odds ratios (ORs) with 95% confidence intervals were estimated by logistic regression models. ^b^ The adjusted odds ratios (AOR) with 95% confidence intervals were estimated by multiple logistic regression models after controlling for alcohol and tobacco consumption.

**Table 4:** The *MACC1* SNPs genotype frequencies of people who didn’t used to chew betel quid.

Variable	Controls (N = 1001) *n* (%)	Patients (N = 196) *n* (%)	OR ^a^ (95% CI)	AOR ^b^ (95% CI)
rs3095007				
GG	840 (83.9%)	169 (86.2%)	1.00	1.00
GT	154 (15.4%)	27 (13.8%)	0.871 (0.561–1.355)	0.876 (0.559–1.372)
TT	7 (0.7%)	0 (0.0%)	---	---
GT + TT	161 (16.1%)	27 (13.8%)	0.834 (0.537–1.294)	0.835 (0.534–1.306)
rs1990172				
GG	755 (75.4%)	144 (73.5%)	1.00	1.00
GT	234 (23.4%)	48 (24.5%)	1.075 (0.752–1.539)	1.075 (0.747–1.549)
TT	12 (1.2%)	4 (2.0%)	1.748 (0.556–5.495)	2.036 (0.631–6.569)
GT + TT	246 (24.6%)	52 (26.5%)	1.108 (0.782–1.570)	1.117 (0.783–1.593)
rs4721888				
GG	514 (51.3%)	95 (48.5%)	1.00	1.00
GC	417 (41.7%)	84 (42.9%)	1.090 (0.791–1.502)	1.118 (0.806–1.551)
CC	70 (7.0%)	17 (8.7%)	1.314 (0.741–2.331)	1.311 (0.729–2.358)
GC + CC	487 (48.7%)	101 (51.5%)	1.122 (0.826–1.524)	1.147 (0.839–1.567)
rs975263				
TT	685 (68.4%)	135 (68.9%)	1.00	1.00
TC	292 (29.2%)	57 (29.1%)	0.990 (0.706–1.390)	1.029 (0.728–1.453)
CC	24 (2.4%)	4 (2.0%)	0.846 (0.289–2.477)	0.885 (0.297–2.642)
TC + CC	316 (31.6%)	61 (31.1%)	0.979 (0.704–1.363)	1.018 (0.727–1.426)
rs3735615				
GG	735 (73.4%)	148 (75.5%)	1.00	1.00
GC	248 (24.8%)	46 (23.5%)	0.921 (0.642–1.321)	0.899 (0.623–1.299)
CC	18 (1.8%)	2 (1.0%)	0.552 (0.127–2.404)	0.598 (0.135–2.643)
GC + CC	266 (26.6%)	48 (24.5%)	0.896 (0.629–1.277)	0.880 (0.613–1.263)

^a^ The odds ratios (ORs) with 95% confidence intervals were estimated by logistic regression models. ^b^ The adjusted odds ratios (AORs) with 95% confidence intervals were estimated by multiple logistic regression models after controlling for alcohol and tobacco consumption.

**Table 5:** The effect of *MACC1* SNPs genotype frequencies of 715 oral cancer patients who chewed betel quid on the clinicopathological parameters.

Parameters	*MACC1* rs975263 (Betel Quid Chewers)			
	TT (*n* = 488) *n* (%)	TC + CC (*n* = 227) *n* (%)	OR ^a^ (95% CI)	*p* Value
Clinical Stage				
I/II	238 (48.8%)	118 (52.0%)	1.00	*p* = 0.424
III/IV	250 (51.2%)	109 (48.0%)	0.879 (0.642–1.205)	
Tumor size				
≤T2	264 (54.1%)	140 (61.7%)	1.00	*p* = 0.057
>T2	224 (45.9%)	87 (38.3%)	0.732 (0.531–1.010)	
Lymph node metastasis				
No	346 (70.9%)	143 (63.0%)	1.00	*p* = 0.034 *
Yes	142 (29.1%)	84 (37.0%)	1.431 (1.026–1.996)	
Cell differentiation				
Well	72 (14.8%)	34 (15.0%)	1.00	*p* = 0.937
Moderate/Poor	416 (85.2%)	193 (85.0%)	0.982 (0.631–1.529)	

^a^ The odds ratio (OR) with their 95% confidence intervals were estimated by logistic regression models. * *p* value < 0.05 as statistically significant.

## Data Availability

The data presented in this study are available on request from the corresponding author.

## Supplementary Materials

The following are available online at https://www.mdpi.com/article/10.3390/jpm11060490/s1. Table S1: The context sequence of five MACC1 SNPs in the study. Table S2: The MACC1 SNPs genotype frequencies of people who were used to cigarette smoking. Table S3: The MACC1 SNPs genotype frequencies of people who were used to alcohol drinking. Figure S1: Representative TaqMan assay for MACC1 rs4721888 genotyping.
