# Supplementary material for: Effect of MACC1 Genetic Polymorphisms and Environmental Risk Factors in the Occurrence of Oral Squamous Cell Carcinoma
Source: J Pers Med. 2021 May 31;11(6):490. doi: 10.3390/jpm11060490 (PMC8228283; doi:10.3390/jpm11060490)
Supplement: Supplementary file 1 [file jpm-11-00490-s001.zip › jpm-1202722-supplementary.pdf]

Table S1. The context sequence of five MACC1 SNPs in the study.

Variable	Assay ID	Context Sequence
**rs3095007**	C__27452671_10	TACAATGCACTGTGCTAATTTAATG[A/C] CCCCCTTAACTTAATTACATGATTA
**rs1990172**	C___2632417_10	TGTTTCCAGGCATGATAATTAAATA[A/C] ATCCAAAAATTGTCTCATAAGGTGG
**rs4721888**	C__27869756_10	CCTGTAATATTGCAACTTTTTGAGA[C/G] TTTTCCAGCTTCCATGTCAATCAAA
**rs975263**	C___2632422_20	CTTCTGCAAATAGCCTGGCAGATTC[G/A] AGAGTCTTTTTAGGTTTGGGGTTGG
**rs3735615**	C__27489506_10	TGACTGAAGGTCTTGTAACACATCT[C/G] TGTAGTTATTTCCATAGTGAGCACT

Table S2. The MACC1 SNPs genotype frequencies of people who were used to cigarette smoking.

Variable	Controls (N=636) n (%)	Patients (N=809) n (%)	OR[a] (95% CI)	AOR[b] (95% CI)
**rs3095007**				
GG	530 (83.3%)	678 (83.8%)	1.00	1.00
GT	100 (15.7%)	127 (15.7%)	0.993 (0.746-1.321)	0.962 (0.680-1.362)
TT	6 (0.9%)	4 (0.5%)	0.521 (0.146-1.856)	0.373 (0.084-1.657)
GT+TT	106 (16.7%)	131 (16.2%)	0.966 (0.730-1.278)	0.924 (0.658-1.298)
**rs1990172**				
GG	464 (73.0%)	609 (75.3%)	1.00	1.00
GT	166 (26.1%)	183 (22.6%)	0.840 (0.659-1.070)	0.805 (0.600-1.081)
TT	6 (0.9%)	17 (2.1%)	2.159 (0.845-5.518)	2.259 (0.731-6.980)
GT+TT	172 (27.0%)	200 (24.7%)	0.886 (0.699-1.123)	0.852 (0.639-1.137)
**rs4721888**				
GG	346 (54.4%)	410 (50.7%)	1.00	1.00
GC	247 (38.8%)	338 (41.8%)	1.155 (0.929-1.436)	1.289 (0.989-1.681)
CC	43 (6.8%)	61 (7.5%)	1.197 (0.790-1.814)	1.534 (0.923-2.549)
GC+CC	290 (45.6%)	399 (49.3%)	1.161 (0.943-1.430)	1.323 (0.953-1.726)
**rs975263**				
TT	442 (69.5%)	556 (68.7%)	1.00	1.00
TC	178 (28.0%)	230 (28.4%)	1.027 (0.814-1.296)	1.062 (0.801-1.409)
CC	16 (2.5%)	23 (2.9%)	1.143 (0.596-2.189)	1.000 (0.458-2.180)
TC+CC	194 (30.5%)	253 (31.3%)	1.037 (0.828-1.298)	1.057 (0.804-1.389)
**rs3735615**				
GG	450 (70.8%)	581 (71.8%)	1.00	1.00
GC	173 (27.2%)	217 (26.8%)	0.972 (0.768-1.228)	0.874 (0.658-1.162)
CC	13 (2.0%)	11 (1.4%)	0.655 (0.291-1.477)	0.440 (0.165-1.177)
GC+CC	186 (29.2%)	228 (28.2%)	0.949 (0.755-1.194)	0.842 (0.637-1.112)

[a]The odds ratio (OR) with their 95% confidence intervals were estimated by logistic regression models.

[b]The adjusted odds ratio (AOR) with their 95% confidence intervals were estimated by multiple logistic regression models after controlling for chew betel quid and alcohol consumption.

Table S3. The MACC1 SNPs genotype frequencies of people who were used to alcohol drinking.

Variable	Controls (N=237) n (%)	Patients (N=490) n (%)	OR[a] (95% CI)	AOR[b] (95% CI)
**rs3095007**				
GG	196 (82.7%)	412 (84.1%)	1.00	1.00
GT	38 (16.0%)	76 (15.5%)	0.951 (0.622-1.455)	0.847 (0.514-1.395)
TT	3 (1.3%)	2 (0.4%)	0.317 (0.053-1.913)	0.182 (0.027-1.234)
GT+TT	41 (17.3%)	78 (15.9%)	0.905 (0.598-1.370)	0.785 (0.483-1.276)
**rs1990172**				
GG	171 (72.2%)	376 (76.7%)	1.00	1.00
GT	63 (26.6%)	105 (21.4%)	0.758 (0.528-1.087)	0.669 (0.437-1.024)
TT	3 (1.3%)	9 (1.9%)	1.364 (0.365-5.103)	0.717 (0.173-2.965)
GT+TT	66 (27.8%)	114 (23.3%)	0.786 (0.552-1.118)	0.672 (0.443-1.019)
**rs4721888**				
GG	131 (55.3%)	254 (51.8%)	1.00	1.00
GC	95 (40.1%)	199 (40.6%)	1.080 (0.782-1.492)	1.127 (0.770-1.648)
CC	11 (4.6%)	37 (7.6%)	1.735 (0.857-3.512)	2.023 (0.889-4.602)
GC+CC	106 (44.7%)	236 (48.2%)	1.148 (0.841-1.568)	1.215 (0.842-1.754)
**rs975263**				
TT	165 (69.6%)	341 (69.6%)	1.00	1.00
TC	66 (27.8%)	136 (27.8%)	0.997 (0.704-1.412)	0.922 (0.613-1.389)
CC	6 (2.6%)	13 (2.6%)	1.048 (0.391-2.807)	0.799 (0.258-2.476)
TC+CC	72 (30.4%)	149 (30.4%)	1.001 (0.715-1.403)	0.911 (0.613-1.354)
**rs3735615**				
GG	166 (70.0%)	355 (72.4%)	1.00	1.00
GC	65 (27.4%)	125 (25.5%)	0.899 (0.633-1.278)	0.819 (0.541-1.241)
CC	6 (2.6%)	10 (2.1%)	0.779 (0.279-2.180)	0.423 (0.138-1.294)
GC+CC	71 (30.0%)	135 (27.6%)	0.889 (0.632-1.251)	0.775 (0.519-1.159)

[a]The odds ratio (OR) with their 95% confidence intervals were estimated by logistic regression models.

[b]The adjusted odds ratio (AOR) with their 95% confidence intervals were estimated by multiple logistic regression models after controlling for chew betel quid and alcohol consumption.

Figure S1

MACC1 rs4721888

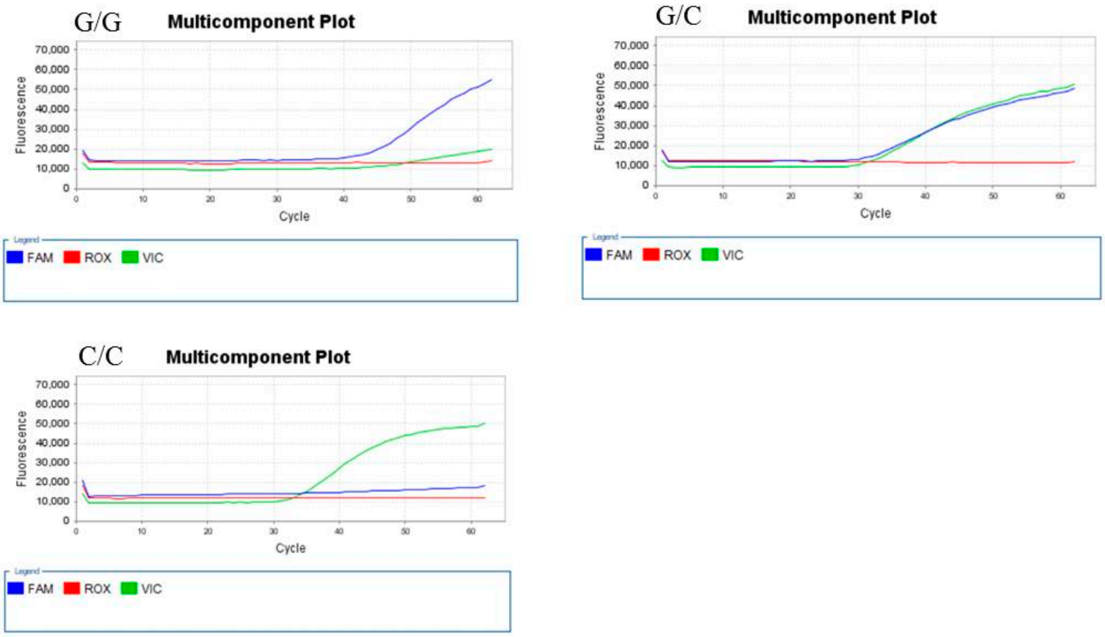

Figure S1: Representative TaqMan assay for MACC1 rs4721888 genotyping.
